# The Protective Effect of New Compound XH-103 on Radiation-Induced GI Syndrome

**DOI:** 10.1155/2018/3920147

**Published:** 2018-07-04

**Authors:** Yinping Dong, Ying Cheng, Qinlian Hou, Jing Wu, Deguan Li, Hongqi Tian

**Affiliations:** Tianjin Key Laboratory of Radiation Medicine and Molecular Nuclear Medicine, Institute of Radiation Medicine, Chinese Academy of Medical Science and Peking Union Medical College, Tianjin 300192, China

## Abstract

**Background:**

Radiation-induced intestinal injury is one of the side effects in patients receiving radiotherapy. The aim of the present study was to investigate the protective effect of XH-103 on radiation-induced small intestinal injury and to explore its mechanism.

**Methods:**

C57BL/6N mice were irradiated and treated with XH-103. Firstly, the survival rate of mice exposed to 9.0 Gy and 11.0 Gy total body irradiation (TBI) was examined. Subsequently, at 3.5 d after IR, the small intestinal morphological changes were examined by HE. The numbers of crypt cells, the villus height, the expression of Ki67 and Lgr5, and the apoptotic cells in the intestinal crypts were examined by immunohistochemistry. Furthermore, the expression of p53 and Bax was analyzed by WB.

**Results:**

Compared to the irradiation group, XH-103 improved the mice survival rate, protected the intestinal morphology of mice, decreased the apoptotic rate of intestinal crypt cells, maintained cell regeneration, and promoted crypt proliferation and differentiation. XH-103 also reduced the expression of p53 and Bax in the small intestine compared to the IR group.

**Conclusion:**

These data demonstrate that XH-103 can prevent radiation-induced intestinal injury, which is beneficial for the protection of radiation injuries.

## 1. Introduction

Currently, radiation therapy is widely used in a variety of cancer treatments. The small intestine is one of the most sensitive organs to ionizing radiation (IR) in the human body. High doses of ionizing radiation induce acute damage to epithelial cells of the intestines and produce death within 10 days reflecting toxicity to the gastrointestinal (GI) tract [1]. In contrast to radiation-induced bone marrow damage which can be prevented by bone marrow transplantation, there are no approved methods to prevent or treat radiation-induced gastrointestinal syndrome (RIGS) [2]. The main symptoms of radiation-induced intestinal damage include anorexia, vomiting, diarrhea, dehydration, systemic infection, and in extreme cases, septic shock and death [3]. Radiation-induced intestinal damages seriously affect the treatment of patients with abdominal or pelvic tumors, reducing the quality of life of patients. Therefore, the development of efficient radiological intestinal drugs is an important area of radiation protection.

Natural antioxidant agents and aminothiol compounds have been intensively investigated for the radiation protection application [4–6]. Flavonoids belong to natural antioxidant agents that can suppress the formation of free radicals and have antioxidant effects. However, it has disadvantage of poor stability and bioavailability [7]. Aminothiol compound possesses good efficacy profile but short half-life and high toxicity [8].

**Scheme 1:**
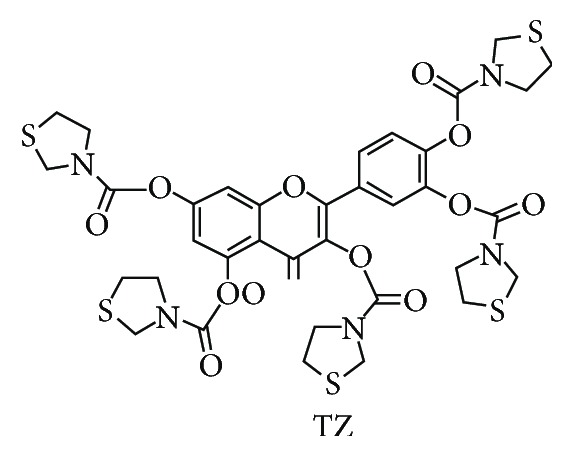


In this study, we take the natural antioxidation agent quercetin which belongs to flavonoid family derived from plants as a lead compound and modify the molecular structure. At the same time, we also try to combine the aminothiol analogue and the natural antioxidation agent together with different linkers in order to retain the efficacy of aminothiol and the safety property of natural antioxidation agent, respectively. The quercetin group and aminothiol group can modulate pharmacokinetic (PK) profile mutually. Finally, we designed and synthesized the compound TZ (XH-103) (Scheme 1).

In this study, to define the effect of XH-103 on intestinal repair and regeneration following radiation injury, we used a mouse model of radiation-induced intestinal damage by exposure to 9.0 Gy total body irradiation (TBI). We found that the XH-103 could improve the survival rate of mice and intestinal epithelium cells (IECs). We also found that the crypt-villous structure injuries of the small intestines and the apoptosis of IECs induced by TBI were mitigated by XH-103. And XH-103 could protect the proliferation and differentiation of intestinal stem cells (ISCs). Here, we evaluated the possibility and mechanisms of the radiation protective effects of XH-103 on radiation-induced intestinal injury.

## 2. Materials and Methods

### 2.1. Synthesis of Thiazolidine-3-carbonyl Chloride

To a solution of bis(trichloromethyl) carbonate (4.79 g, 53.82 mmol) (Energy Chemical, Shanghai, China) in anhydrous tetrahydrofuran (35 mL) were added thiazolidine (4 g, 44.94 mmol) (Energy Chemical, Shanghai, China) in parts and triethylamine (8.1 mL) dropwise, the mixture was stirred under nitrogen atmosphere and in ice water bath for 1 h and then at room temperature for 10 h. The reaction mixture was filtered, and the residue was washed with dichloromethane (10 mL) for three times. The filtrate phase was combined and evaporated in vacuo. The resultant residue was used directly for the next step.

### 2.2. Synthesis of 2-(3,4-Bis((thiazolidine-3-carbonyl)oxy)phenyl)-4-oxo-4H-chromene-3,5,7-triyl Tris(thiazolidine-3-carboxylate)

To a solution of 2-(3,4-dihydroxyphenyl)-3,5,7-trihydroxy-4H-chromen-4-one (1.6 g, 5.3 mmol) (Shuya Chemical Science and Technology, China) in DMF (30 mL) were added triethylamine (7 mL), 4-dimethylaminopyridine (192 mg, 1.57 mol), and thiazolidine-3-carbonyl chloride, the mixture was stirred under 0°C for 1 h and at room temperature for 10 h. The reaction mixture was poured into MeOH/H_2_O/DMSO 8/1.5/0.5 *v*/*v*/*v* (500 mL), and then water was added dropwise until the product precipitated. The residue was then filtered and washed with water (20 mL) for three times. The filter cake was then dried under vacuo to give the title compound as a yellow solid (3.35 g, 72%): LC-MS: *R*_T_ = 4.5 min; [M + H]^+^ = 879.20, calculated 878.99, ^1^H NMR (400 MHz, DMSO) *δ* ppm 7.95–7.85 (m, 2H), 7.68 (s, 1H), 7.58 (d, *J* = 8.3 Hz, 1H), 7.25 (s, 1H), 4.58 (dd, *J* = 52.9, 22.8 Hz, 10H), 3.97–3.65 (m, 10H), and 3.14 (d, *J* = 20.2 Hz, 10H).

### 2.3. Animals

Male C57BL/6 mice (8–10 weeks) were purchased from Beijing HFK Bioscience Co. Ltd. (Beijing, China). Animals were bred in the certified animal facility in the Institute of Radiation Medicine (IRM) of Chinese Academy of Medical Sciences (CAMS).

### 2.4. Ethics Approval and Consent to Participate

All experimental procedures were carried out in accordance with the NIH Guidelines for the Care and Use of Laboratory Animals and were approved by the Institutional Animal Care and Use Committee of the Institute of Radiation Medicine (IRM), Chinese Academy of Medical Sciences (CAMS) (Permit Number 2017053). The animals were cared for in accordance with the dictates of the National Animal Welfare Law of China.

### 2.5. Irradiation and Treatment

The mice were exposed to ionizing radiation by using a ^137^Cs source following an Exposure Instrument Gammacell-40 (Atomic Energy of Canada Lim, Chalk River, ON, Canada) at a dose rate of 1.0 Gy/min. The mice were exposed to 9.0 Gy and 11.0 Gy TBI in the survival experiments (*n* = 10).

In the remaining experiments, the animals were divided randomly into three groups (*n* = 5): (a) control, (b) IR + vehicle, and (c) IR + 103, and received 9.0 Gy TBI. XH-103 was dissolved in 4% DMSO; 96% of PEG400 was added after heating for a final concentration of 20 mg/mL. Individual mice in the IR + 103 group received a dose of 200 mg/kg XH-103 administered by gavage 1 hour before irradiation. The mice of the groups control and IR + vehicle were treated with vehicle similarly to the procedure described for the XH-103 treatment.

### 2.6. Histological Analysis

At three days after IR, the mice were sacrificed, and the small intestines were collected and stained with hematoxylin-eosin (H&E) and analyzed under a microscope. For morphological analysis, six circular transverse sections were analyzed per mice in a blind manner from coded digital HE-stained photographs to measure the villi length and crypt number by using the ImageJ 1.37 software.

### 2.7. Immunohistochemistry Analysis

The 4 *μ*m-thick sections of paraffin-embedded small intestine sections were dewaxed and rehydrated with citrate buffer. Then, the sections were boiled in 10 mM/L citrate buffer solution (pH 9.0) for antigen retrieval according to the standard procedures. After antigen retrieval, the sections were incubated with serum for 1 h at room temperature to block nonspecific antigen-binding sites and then with anti-Lgr5 antibody (1 : 50 dilution, Abcam, Cambridge, MA, USA), anti-Ki67 antibody (1 : 300 dilution, Novus a biotechne brand), anti-lysozyme (1 : 800 dilution, Abcam, Cambridge, MA, USA), or anti-villi (1 : 800 dilution, Abcam, Cambridge, MA, USA) overnight at 4°C. Sections were then incubated in secondary antibody for 30 min at 37°C. Positive cells were detected using DAB kit (Sigma-Aldrich). The images were captured, and positive staining was quantified objectively by the IPP software as described previously in a blinded fashion.

### 2.8. TUNEL Assay

The 3 *μ*m-thick sections were treated according to the manufacturer's protocols (Roche, Mannheim, Germany). Sections were analyzed by light microscope.

### 2.9. Isolation of Intestinal Crypt Cells

The method of isolating intestinal crypts was described [9, 10]. Briefly, after flushing with ice-cold PBS, the small intestines were chopped into small pieces and then placed into cold PBS containing 2 mM EDTA for 30 min. After rinsing twice with ice-cold PBS, the fragments were resuspended in cold dissociation buffer. The solution was filtered through a 70 *μ*m strainer to remove the villus fraction and collect the crypt fraction. The crypt fraction was centrifuged to isolate the single cells.

### 2.10. Western Blot Analysis

Protein was extracted from small intestinal crypt cells with ice-cold lysis buffer (Solarbio Science and Technology, Beijing, China). Protein concentration was quantified using the bicinchoninic acid protein assay kit (Beyotime, Shanghai, China), and equal amounts of protein were resolved by SDS-PAGE gel. The blocked membrane was incubated using anti-Bax antibody (1 : 1000 dilution, Ruiying Bio, Suzhou, China) and antibodies against *β*-tubulin (1 : 2500 dilution, Proteintech, Wuhan, China) overnight at 4°C. Then, the membranes were incubated in suitable horseradish peroxide-conjugated secondary antibody for 1-2 hours at room temperature. Finally, the chemiluminescent substrate (Millipore Corporation, Billerica, MA 01821, USA) is used to detect protein.

### 2.11. Immunofluorescence Analysis

The paraffin-embedded sections of the small intestine were subjected to antigen retrieval as described above and then washing thoroughly with PBS. The sections were blocked with 5% goat serum for 30 min at room temperature and then incubated with anti-caspase-8 (1 : 100 dilution, CST, MA, USA), anti-caspase-9 (1 : 1000 dilution, CST, MA, USA), anti-*γ*H2AX (1 : 1000 dilution, BD biosciences, NJ, USA), and anti-p53 (1 : 1000 dilution, Ruiying Bio, Suzhou, China) overnight at 4°C. After washing with PBS, the sections were incubated in the secondary antibody for 40 min at 37°C avoiding light. The sections were finally sealed with DAPI-containing sealing agent. The images were captured by laser scanning confocal microscope (LSCM).

### 2.12. Statistical Analysis

Mice survival curves were analyzed by Kaplan-Meier method using GraphPad Prism 6.0 software for Mac. The data were expressed as mean ± standard deviation (SD). Analysis of variance (ANOVA) test was used to analyze differences among the groups, and *t*-test was used to analyze the difference between the two groups.

## 3. Results

### 3.1. Synthesis and Characterization of XH-103

**Figure 1:**
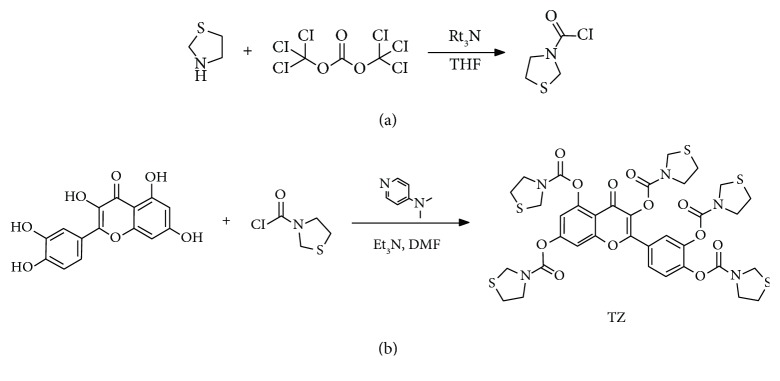
Synthesis and structure of TZ (XH-103). (a) The thiazolidine was reacted with bis(trichloromethyl) carbonate with triethylamine as base to prepare thiazolidine-3-carbonyl chloride. (b) Thiazolidine-3-carbonyl chloride was coupled with quercetin in the presence of triethylamine and 4-dimethylaminopyridine to afford the product TZ.

Based on design concept, we designed and prepared the compound XH-103, of which the synthetic routes were shown in Figure 1. The synthetic procedures were depicted in Supplementary Materials (available here). Firstly, the thiazolidine was reacted with bis(trichloromethyl) carbonate with triethylamine as base to prepare thiazolidine-3-carbonyl chloride. Then, the as-prepared intermediate was further coupled with quercetin molecule in the presence of triethylamine and 4-dimethylaminopyridine to afford the 2-(3,4-bis((thiazolidine-3-carbonyl) oxy)phenyl)-4-oxo-4H-chromene −3,5,7-triyl tris(thiazolidine-3-carboxylate) (XH-103) as products with the isolation yield of 72%. The structure was characterized by NMR and ESI-MS. The ^1^H NMR and ESI-MS spectrum of XH-103 is shown in Figures S1a and S1b. The results indicated that the new compound XH-103 was successfully prepared with facile synthetic approach.

### 3.2. XH-103 Improves the Survival Rate of Mice after TBI

**Figure 2:**
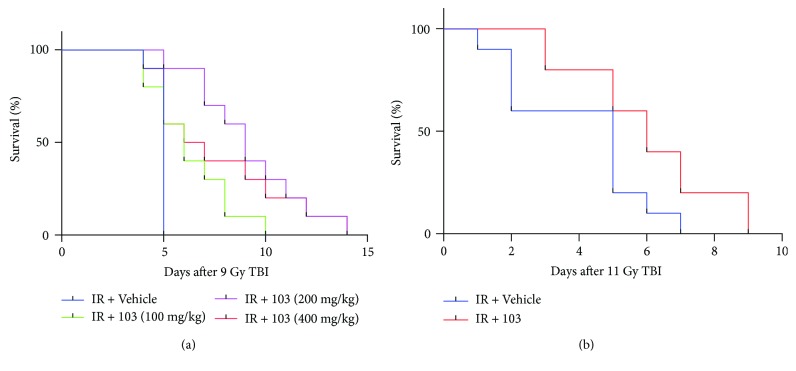
XH-103 enhances the survival rate of mice after TBI. Kaplan-Meier survival analysis of mice exposed to 9.0 Gy or 11.0 Gy TBI. (a) Three doses of XH-103-treated mice show reduced mortality following lethal doses of TBI (9.0 Gy) within 13 days, compared with IR + vehicle group 100% mortality within 5 days (*p* < 0.05, *n* = 10 per group). (b) The Kaplan-Meier survival curve of vehicle- and XH-103-treated mice (*p* < 0.05, *n* = 10 per group) after 11.0 Gy total body irradiation. The data were expressed as the percent of surviving mice.

To determine the protective effect of XH-103 on mice exposure to radiation, we first observed the survival rate of mice after 9.0 Gy TBI (Figure 2(a)). The mice were treated with XH-103 in three dosages (100 mg/kg, 200 mg/kg, and 400 mg/kg); we found that all doses could improve the survival rate of mice compared with the vehicle-treated group. In the following study, there was 80% mortality in vehicle-treated mice within 6 days of 11.0 Gy TBI (Figure 2(b)), compared with XH-103-treated mice having 60% survival, suggesting that XH-103 may have a protective effect on RIGS in mice. These results indicate that XH-103 effectively mitigates the TBI-induced lethality in mice.

### 3.3. XH-103 Reduces the Damages of Intestinal Morphology in Mice after TBI

**Figure 3:**
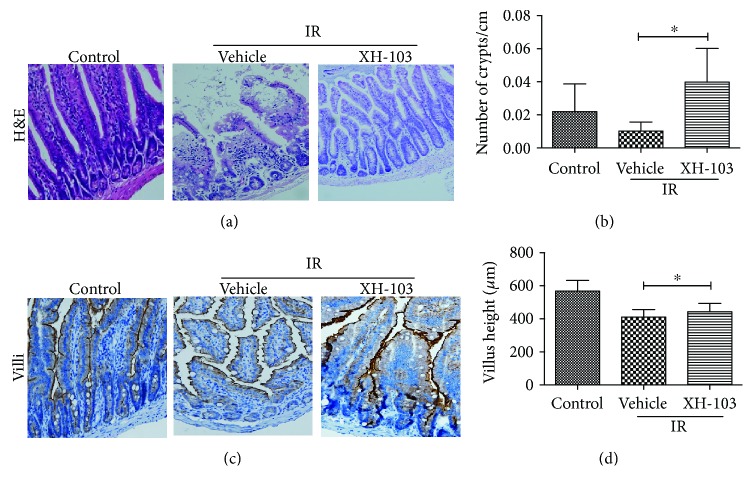
XH-103 protects the intestinal morphology of mice after TBI. (a) Representative images showing the structure in cross sections of the small intestine with H&E stain. (b) Histogram showing the number of crypts. (c) Immunohistochemistry images showing the expression of villi. (d) Histogram demonstrating villus length in intestinal section from the control group, NS-treated group, and XH103-treated group. The results are represented as mean ± SEM, *n* = 5 mice per group. ^∗^ *p* < 0.05. Scale bar: 100 *μ*m and 50 *μ*m.

To determine the effect of XH-103 on radiation-induced intestinal injuries, the morphological changes of mouse small intestine are shown in Figure 3. At 3 d after 9.0 Gy TBI, the irradiated mice showed significantly shorter villous length and fewer crypts than the control group (*p* < 0.05). In comparison to mice in the IR group, XH-103-treated mice showed more survival crypts and increased villus height (*p* < 0.05). The expression of villi^+^ enterocyte was also decreased by IR (Figure 3(c)) then was significantly increased by XH-103 compared to mice in the IR group. These results indicate that XH-103 treatment can prevent postradiation damage of the intestinal crypt-villus structure of mice.

### 3.4. XH-103 Enhances Lgr5^+^ ISC Survival and Maintains the Regeneration of Intestinal Cells after TBI

**Figure 4:**
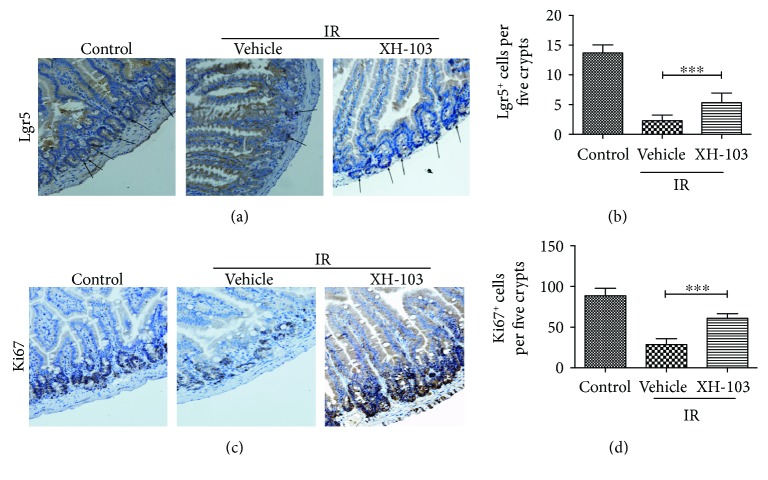
XH-103 increases the proliferation and differentiation of the Lgr5^+^ small intestine after TBI. The small intestinal sections were analyzed by IHC. (a) Photomicrograph of Lgr5 immunostaining section of the control, IR + vehicle, and IR+ 103 group. (b) Histogram showing Lgr5-positive cells that were quantified in five crypts per section. (c) Immunostaining images showing quantitative analysis of Ki67 expression of intestinal crypts. (d) Histogram demonstrating Ki67-positive cells that were counted in five crypts per section. The results are represented as mean ± SEM, *n* = 5 mice per group. ^∗∗∗^ *p* < 0.005. Scale bar: 50 *μ*m.

To evaluate the effect of XH-105 on the proliferation and differentiation ability of crypt cells, Lgr5 and Ki67 were identified by immunohistochemistry staining. Lgr5^+^ intestinal stem cells are indispensable for intestinal regeneration following radiation [11]. The numbers of Lgr5^+^ ISCs were significantly increased in XH-103-treated mice than in the IR group (Figures 4(a) and 4(b)). Similarly, the numbers of Ki67^+^-positive cells in XH-103-treated mice were also markedly higher than those of mice in the IR group (Figures 4(c) and 4(d)). These results indicate that XH-103 is helpful to maintain differentiation and proliferation ability of intestinal crypt cells.

### 3.5. XH-103 Decreases Apoptosis of the Small Intestine after TBI

**Figure 5:**
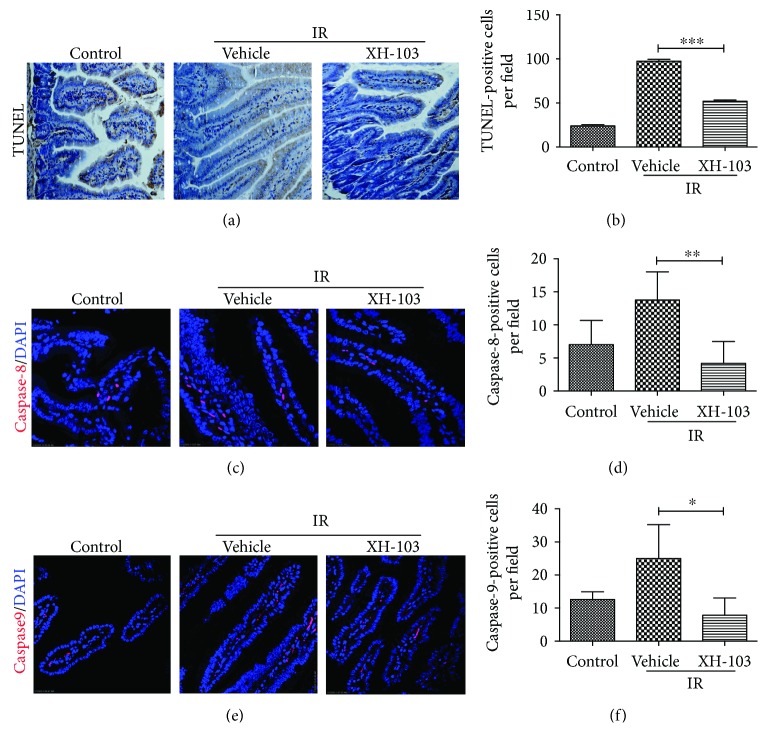
XH-103 reduces the apoptosis of the small intestine after TBI. (a) Apoptosis was assayed by TUNEL staining. (b) The number of TUNEL-positive cells was quantified per field. The paraffin-embedded sections of the small intestine were analyzed by immunofluorescence. (c) Representative DAPI and caspase-8-staining images of the small intestine (red, caspase-8; blue, DAPI). (d) Caspase-8-positive cells in a single field of view were quantified. (e) Photomicrograph of caspase-9-staining images of the small intestine (red, caspase-9; blue, DAPI). (f) Bar graph showing quantitative analysis of caspase-9-positive cells per field of view. The results are represented as mean ± SEM, *n* = 5 mice per group. ^∗^ *p* < 0.05, ^∗∗^ *p* < 0.01, ^∗∗∗^ *p* < 0.005. Scale bar: 50 *μ*m and 10 *μ*m.

To analyze the role of XH-103 in small intestine apoptosis after TBI, we evaluated the apoptosis by TUNEL assay (Figures 5(a) and 5(b)). Compared to the control, more apoptosis cells were observed in the IR group, while XH-103 decreased the apoptosis cells. To further validate our observations, we also determined caspase-8 and caspase-9 expression in small intestine (Figures 5(c) and 5(e)). The similar results were observed. The expressions of caspase-8 and caspase-9 in the XH-103 group were decreased than those in the IR group. These data suggest that XH-103 treatment could decrease the apoptosis cells and protect the irradiation-induced intestinal injuries.

### 3.6. XH-103 Attenuates DNA Damage of the Small Intestine after TBI

**Figure 6:**
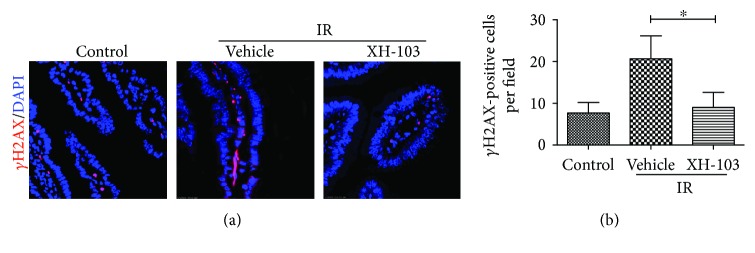
XH-103 attenuates DNA damage of mice after TBI. The small intestines of control mice, vehicle-treated mice, and XH-103-treated mice were obtained at 3 d after 9.0 Gy TBI. (a) Representative immunofluorescence images for the expression of *γ*H2AX of the small intestines (red, γH2AX; blue, DAPI). (b) Histogram demonstrating quantitative analysis of γH2AX-positive cells per view field. The results are represented as mean ± SEM, *n* = 5 mice per group. ^∗^ *p* < 0.05. Scale bar: 10 *μ*m.

To determine whether XH-103 treatment could reduce TBI-induced DNA damage, we analyzed the expression of histone H2AX phosphorylation, which has been widely used as a marker for DNA double-strand breaks (DSBs). As demonstrated in Figure 6, there was an increase in *γ*H2AX of intestinal sections from the IR group compared with the control group. XH-103 treatment decreased H2AX phosphorylation in intestinal sections compared with vehicle-treated mice after 9.0 Gy TBI. The result indicated that XH-103 could reduce IR-induced DNA damage to the small intestine.

### 3.7. XH-103 Protects the Small Intestine against Radiation-Induced Injury at Least in Part via p53 Pathway

**Figure 7:**
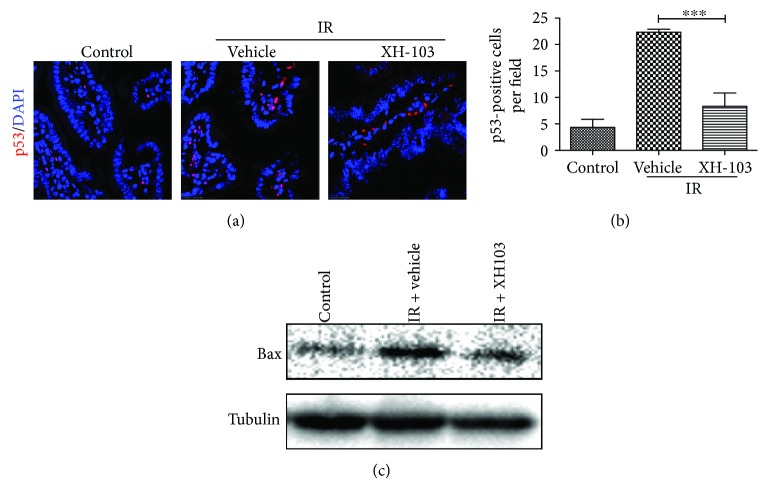
XH-103 decreases the expression of p53 and Bax after TBI. The small intestinal sections of the control, IR + vehicle, and IR+ 103 mice were gained at 3 d after 9.0 Gy TBI. (a) Representative immunofluorescence images for the expression of p53 of the small intestines (red, p53; blue, DAPI). (b) Histogram showing quantitative analysis of p53-positive cells per field of view. (c) Western blot for Bax and tubulin in the intestinal crypts from non-IR mice, vehicle-treated mice, and XH-103-treated mice at 3 d after 9.0 Gy TBI. The results are represented as mean ± SEM, *n* = 5 mice per group. ^∗∗∗^ *p* < 0.005. Scale bar: 10 *μ*m.

To investigate the mechanisms on how XH-103 protects against the radiation-induced intestinal injuries, we determine the expression of p53 by immunofluorescence analysis (Figure 7(a)). Radiation activates p53 in the GI epithelium, and p53-mediated apoptosis has been implicated in regulating the intestinal radiation injuries [12, 13]. We also evaluated the expression of *Bax* in the small intestine crypts by Western blotting at 3 d after 9.0 Gy TBI (Figure 7(c)). IR increased the expression of p53 in the small intestine compared with the control group. In contrast, mice treated with XH-103 downregulate the expression of p53 and Bax (Figures 7(b) and 7(c)). These findings suggest that XH-103 protects the small intestine from IR at least by p53 pathway.

## 4. Discussion

The development of the effective method and drug to mitigate the radiation-induced intestinal injuries is an important area in cancer therapy, nuclear accident, and terrorism. Many studies have reported that Chinese herbal medicine or extracts may reduce TBI-induced injuries in the brain, esophagus, and hematopoietic system of irradiated animals [14–18]; the study of protective drugs on IR-induced intestinal injuries still needs to be improved. In the present study, we synthesized a new compound XH-103 and evaluated the protective effects on radiation-induced intestinal injuries.

In this study, we observe that XH-103 improved the survival rate of mice exposed to the lethal dose TBI, which indicates that XH-103 could protect the mice from irradiation. Under physiological conditions, epithelial homeostasis is maintained by proliferative cells in crypts, and the small intestinal crypt cells are particularly sensitive to IR due to their high proliferative rate [19]. The intestinal epithelium is one of the most rapidly self-renewing organizations in mammals, which is continuously renewed by intestinal epithelium stem cells (IESCs) located in the crypts. Intestinal epithelium cell renewal is identified by expression of Lgr5 [20, 21]. We found that the numbers of Lgr5^+^ intestinal stem cells were increased in the XH-103 group after 9.0 Gy TBI, and the Lgr5^+^ intestinal stem cells differentiated into more Paneth cells and villus cells. Thus, XH-103 may play the protective role on IR-induced intestinal injuries by improving the proliferation and differentiation of Lgr5^+^ intestinal stem cells. The increased expression of Ki67, another proliferative marker in the small intestine, in the XH-103 mice also suggested the protective effects of XH-103 on the intestinal radiation injury. After radiation, various degrees of villus blunting and fusion, villous epithelial cell attenuation and hypertrophy, and severe loss of crypts may occur, leading to destruction of epithelial cell homeostasis and epithelial integrity [13]. Incomplete epithelial cells cannot easily maintain intestinal absorption and defense functions. Our results demonstrated that the intestinal crypt-villus structure from the XH-103-treated mice was well preserved after 9.0 Gy TBI. These results indicate that XH-103 may have a protective effect on irradiation-induced intestinal injury.

Apoptosis is programmed cell death that involves the controlled breakdown of intracellular components. Many studies have shown that IR induced tissue damage, such as small intestinal injuries, with increasing apoptosis cells [22–24]. Caspases are a family of genes important for maintaining homeostasis through regulating apoptosis and inflammation [25, 26]. Caspases involved in apoptosis have been subclassified by their mechanism of action, initiator caspase (caspase-8 and -9) and executioner caspase (caspase-3, 6, and 7). In this study, we investigated that treatment with XH-103 reduces the apoptosis cells compared with the IR group. The expression of caspase-8 and caspase-9 in the small intestines of XH-103 was also decreased compared to that of the IR group. These results show the inhibitory effects of XH-103 on radiation-induced apoptosis.

Radiation induces DNA damages [27] and destroys the expression of proteins in cells [28], activating p53 [23, 27, 29, 30]. H2AX phosphorylation is an indicator for quantifying DNA double-strand breaks [31–33]. In this study, we found that the expression of *γ*H2AX was decreased in the XH-103-treated mice compared with the vehicle-treated mice.

It is well known that p53 activates genes that regulate cell cycle checkpoints, DNA damage and repair, and apoptosis [34–36]. p53 can promote apoptosis through interactions with *Bcl-2* family proteins in the cytoplasm. Studies reported that *Bax^−/−^* and *Bak1^−/−^* mice reduced the epithelial cell apoptosis exposure to irradiation [37, 38]. Therefore, we examined whether XH-103 inhibits apoptosis by p53 pathway. For these experiments, treatment with XH-103 could decrease the level of p53 and Bax expression. The data suggested that XH-103 might mitigate the radiation-induced intestinal injuries by p53-dependent apoptosis pathway.

Our studies synthesize a new compound XH-103 and show protective effects of XH-103 against radiation-induced intestinal injury. The results also suggest that XH-103 may attenuate radiation-induced intestinal damage via the p53 pathway. However, XH-103 is a novel compound that needs further optimization.

## Data Availability

The data used to support the findings of this study are available from the corresponding author upon request.

## Supplementary Materials

Supplementary Materials Figure S1a: ^1^H NMR spectra of TZ. ^1^H NMR (400 MHz, DMSO-d6) *δ* ppm 7.95–7.85 (m, 2H), 7.68 (s, 1H), 7.58 (d, *J* = 8.3 Hz, 1H), 7.25 (s, 1H), 4.58 (dd, *J* = 52.9, 22.8 Hz, 10H), 3.97–3.65 (m, 10H), 3.14 (d, *J* = 20.2 Hz, 10H). Figure S1b: LC-MS spectra of TZ. LC-MS method. Experiments performed on a Waters 3100 Mass Detector. Chromatographic column: Agilent, ZORBAX, C18 2.1^∗^50 mm 3.5 *μ*. Flow rate: 0.6 mL/min; mobile phase A: 0.1% formic acid in water; mobile phase B: 100% acetonitrile. Gradient elution program: *t* = 0 min, A% = 10%; *t* = 5.5 min, A% = 95%; *t* = 6.5 min, A% = 95%; *t* = 7 min, A% = 10%; *t* = 10 min, A% = 10%; *RT* = 4.50 min, MS (ESI, positive ion) m/z: 878.15 (M + 1).
